# The Cost of Not Retesting: Human Immunodeficiency Virus Misdiagnosis in the Antiretroviral Therapy “Test-and-Offer” Era

**DOI:** 10.1093/cid/cix341

**Published:** 2017-04-24

**Authors:** Jeffrey W. Eaton, Cheryl C. Johnson, Simon Gregson

**Affiliations:** 1 Department of Infectious Disease Epidemiology, Imperial College London, United Kingdom;; 2 HIV Department, World Health Organization, Geneva, Switzerland; and; 3 Biomedical Research and Training Institute, Harare, Zimbabwe

**Keywords:** HIV testing, misdiagnosis, antiretroviral treatment, cost-benefit analysis.

## Abstract

We compared estimated costs of retesting human immunodeficiency virus (HIV)-positive persons before antiretroviral therapy (ART) initiation to the costs of ART provision to misdiagnosed HIV-negative persons. Savings from averted unnecessary ART costs were greater than retesting costs within 1 year using assumptions representative of HIV testing performance in programmatic settings. Countries should implement re-testing before ART initiation.

New guidelines from the World Health Organization (WHO) recommend antiretroviral therapy (ART) for all people with human immunodeficiency virus (HIV) irrespective of disease status [1]. This recommendation underscores the importance of HIV testing quality to ensure that only people living with HIV are placed on lifelong treatment.

Current WHO guidance recommends independent retesting to verify HIV infection prior to ART initiation, and the WHO have and recently reemphasized this as ART programs have rapidly expanded [2–5]. However, this recommendation is poorly implemented: a 2015 review found that only 2 out of 48 national HIV testing policies stated that retesting should be carried out before ART initiation [6]. Limited uptake of this recommendation may be due to a perceived high cost of retesting, overstretched health workers and system capacity, and uncertainty about how to implement retesting.

The risk of misclassification resulting in initiating HIV-negative people on ART is not hypothetical [7, 8]. In Malawi, 4.6% of people referred for ART in 2015 were subsequently found to be HIV-negative when retested [9]. In Zimbabwe, 77 (3.8%) of 2033 HIV-positive women in testing for prevention of mother-to-child transmission (PMTCT) services were HIV-negative in laboratory tests done for surveillance in 2012 [10]. A multicountry study using program and surveillance reports from testing of pregnant women in programmatic settings yielded estimates for testing algorithm specificity between 98.5% and 99.5% [11].

Misclassification and unnecessarily treating HIV-negative persons has many important ethical, legal, and health system consequences. We examined retesting from a purely economic perspective; and assessed the cost of retesting before ART initiation compared with the cost saving that could be achieved by not providing lifelong ART to misdiagnosed (HIV-negative) persons.

## METHODS

We created a simple model to estimate the cost of HIV testing using WHO recommended serial HIV testing strategies, the expected number of misclassified HIV-negative persons initiated on ART, and the costs of providing lifelong ART to misclassified persons. The model is calibrated to reflect the performance of HIV testing algorithms observed in programmatic settings and HIV testing and treatment costs in a LMIC (Low and Middle Income Countries) setting.

### Human Immunodeficiency Virus Testing Assumptions

For an HIV-positive diagnosis, WHO recommends a serial testing strategy with at least 2 consecutive positive rapid diagnostic tests (RDTs) in high-prevalence settings (>5%) and 3 consecutive positive RDTs in low-prevalence settings (<5%) [5]. Manufacturer specifications stipulating >99% specificity for each RDT would ensure at least 99.99% specificity for both the 2-test and 3-test strategies if each test were independent [12]. To replicate the performance of these testing strategies observed in programmatic settings, we assumed 98% specificity for each RDT. Further, we allowed for systematic errors for consecutive tests to capture the possibility of correlated human errors between otherwise independent RDTs. This is enacted by assuming that if the first test misclassified an HIV-negative person as HIV-positive, the subsequent confirmatory test would have a 20% chance of also misclassifying the individual (in addition to the assumed 98% specificity). Such correlated errors could be attributable to environmental conditions or to user error affecting the outcome of both tests [13]. Overall, these assumptions yielded 99.6% specificity for the 2-test strategy and 99.9% for the 3-test strategy. These levels are consistent with or slightly better than the testing algorithm performance estimated in the multicountry Centers for Disease Control and Prevention (CDC) study [11].

We assumed that retesting by an independent health-worker occurs immediately prior to ART initiation using the same serial RDT strategies as for primary diagnosis, and that test algorithm performance for retesting is the same as for initial testing. Finally, we conservatively assume a 5% correlation in misclassification at initial testing and at retesting.

### Cost Assumptions

We assumed costs representative of typical HIV testing and treatment in LMIC settings. For HIV testing, we assumed a cost of US$8 for the first RDT irrespective of HIV status. For those testing positive, each additional confirmatory RDT and associated counseling was US$6 [14]. These were “fully loaded” costs representative of low-income settings incorporating the costs of commodities, healthcare personnel, supply chain, and above-facility management [15].

Conservatively, we assume that costs for retesting are the same as in initial testing (first test US$8, each confirmatory test US$6). In sensitivity analysis, we alternately assumed that the cost of retesting at ART initiation was only the cost of the additional RDT kits at US$2 per test, because the additional costs of facilities, personnel, and counseling are already borne by the ART program.

The annual cost of providing ART was US$450 [16]. We assumed a 30-year life expectancy after ART initiation and discounted the ART cost by 6% per annum over this period. The discounted cost of lifetime ART for a misclassified HIV-negative person was $6300.

### Analysis

We considered HIV testing in 2 settings: one in which HIV prevalence is 1% using the serial 3-test strategy, and the second with 10% HIV prevalence using the 2-test strategy.

For each setting, in a first scenario (without retesting), we calculated the testing cost per 10000 individuals and the expected number of misclassified HIV-negative persons, who we assume are initiated on lifelong ART under universal ART eligibility. In a second scenario (with re-testing), we calculated the additional cost of retesting and the number of individuals who are still misclassified and initiated on lifelong ART. We compared the total cost of testing and discounted ART costs for misclassified individuals between the 2 scenarios. To estimate how long it takes a health system to recoup the retesting costs in averted ART costs, we calculated the duration within which expected savings from averted unnecessary ART provision became greater than the cost of retesting before ART initiation. We tested the sensitivity of this outcome to variation in the assumed 98% test specificity from 92% to 99%.

## RESULTS

In the setting with an HIV prevalence of 1%, testing 10000 with the 3-test strategy cost $83000. Without retesting, 9 HIV-negative people would be misdiagnosed as positive and initiated on ART for life, costing $58000 in unnecessary ART costs. Retesting all those initially diagnosed HIV-positive would cost $2000 and result in an expected 0.03 HIV-negative persons misclassified and initiated on ART. This reduced the expected ART cost to $186, providing a net saving of $56000 (Table 1).

**Table 1.** Comparison of Retesting Costs and Expected Antiretroviral Therapy Costs for Human Immunodeficiency Virus-Negative persons

	Low Prevalence Example	High Prevalence Example
HIV prevalence among testers	1.0%	10.0%
Serial testing strategy	3-test	2-test
Testing strategy specificity^a^	99.9%	99.6%
Positive predictive value	91.3%	96.2%
Total testing cost^b^	$82628	$87020
Number of HIV–initiated on ART	9.2	38.9
Expected lifetime ART cost for HIV–^c^	$57832	$243399
Retesting specificity	99.7%	98.5%
Positive predictive value (retesting)	99.97%	99.94
Total retesting cost	$2011	$14020
HIV–initiated on ART with retesting	0.03	0.6
Expected lifetime ART cost for HIV–	$186	$3628
Expected savings from retesting	$55634	$225751
Time to recover retesting costs by averted ART costs	0.5 y	0.8 y

Abbreviations: ART, antiretroviral therapy; HIV, human immunodeficiency virus; RDT, rapid diagnostic tests.

^a^Assumes a 98% specificity for each serial RDT and allows a correlation such that, if the first test was false-positive, the subsequent confirmatory test also had a 20% probability of being false-positive due to systemic factors contributing to misdiagnosis.

^b^US$8 for the first RDT and $6 for each additional confirmatory RDT and associated counseling.

^c^Total discounted cost for lifetime ART for an HIV-negative person was $6300.

For 10% HIV prevalence, using the 2-test strategy to test 10000 persons cost $87000. Without retesting, 39 HIV-negative people would be misdiagnosed as positive and initiated on ART with the 2-test strategy, costing $243000 in unnecessary ART costs. Retesting HIV-positive people would cost more, $14000, owing to the larger retesting volume. Retesting reduced the number of misclassified HIV-negative persons to 0.6 with an expected ART cost to $3628, providing net savings of $225000.

Savings from preventing unnecessary ART outstripped the additional expenditure on re-testing within 0.5 years for the 1% HIV prevalence scenario and 0.8 years for the 10% HIV prevalence scenario. When assuming a lower retesting cost of US$2 per RDT, savings on ART were greater than retesting costs within 0.15 to 0.25 years. The finding that averted ART costs quickly overtake retesting costs was robust to a range of values for the probability of misdiagnosis (Supplementary Figure S1), suggesting that retesting before ART initiation will likely remain cost-saving even as the quality of HIV testing improves.

## DISCUSSION

Our estimates suggest that retesting all HIV positive persons before ART initiation quickly becomes cost-saving using assumptions representative of testing algorithm performance and HIV testing and treatment costs typical in LMIC settings. This conclusion rests on 2 observations: first the volume of retesting is low compared to initial testing because only those testing positive are retested; and, second, the high cost of providing ART care compared to the cost of an HIV test. Overall, in both low- and high-HIV prevalence settings the cost of retesting was low compared to the initial testing costs (Table 1) and compared to the costs of providing therapeutic ART to diagnosed HIV-positive persons.

Although our model representations of HIV testing and ART costs are simple, the findings are robust to a range of plausible values due to the large difference between the costs of testing and the costs of ART. Our assumption that retesting with RDTs at the point of ART initiation costs the same as initial testing is probably conservative because much of the costs associated with testing (health facilities, personnel) are already borne by the ART program. The finding that retesting costs are recouped within a matter of months or a few years was also robust to a range of values for testing algorithm specificity, ART cost, and HIV prevalence (Supplementary Appendix S2).

The narrow focus of our retesting analysis on the financial and human resource implications to the health system does not capture a number of other important factors: the potential ethical, personal, and social consequences of incorrect diagnosis and treatment for an HIV-negative person, the quality-of-life implications of unneeded regular treatment and potential associated toxicities, and the potential undermining effects of misdiagnosis for confidence in the health system more widely. For these reasons, our analysis of the benefits of retesting is almost certainly conservative when considered from a broader societal perspective.

Implementation of HIV retesting in specific settings will require more detailed analysis using local procurement costs, supply chain, and service delivery considerations. However, our analysis should motivate national planners and implementing partners to carefully consider retesting before ART initiation as part of the care package as they develop national strategic plans and budgets in response to new universal ART eligibility recommendations.

Different retesting approaches could be considered for verifying diagnoses, including the use of laboratory-based supplementary assays, or point-of-care testing using molecular technologies or viral load tests. However, using the same serial RDT strategies avoids delays and potential loss to follow-up due to transporting specimens and waiting for results from offsite laboratories. It also minimizes the need for new equipment, policies, and staff training that could delay implementation of new ART eligibility guidelines. As with all testing, retesting to verify diagnosis should undergo routine external quality assurance.

Understanding and addressing reasons for HIV misdiagnosis is a public health priority. Meanwhile, countries and implementers should strongly consider routine retesting before ART initiation as they formulate national “test-and-offer” guidelines. This is a key enabler for robust implementation of new policies, is good clinical practice, and is expected to save financial and human resources.

## Supplementary Data

Supplementary materials are available at *Clinical Infectious Diseases* online. Consisting of data provided by the authors to benefit the reader, the posted materials are not copyedited and are the sole responsibility of the authors, so questions or comments should be addressed to the corresponding author.

## Supplementary Material

cost_of_not_retesting_R1_appendix_SUBMITTED

## Notes

***Acknowledgments.*** We thank Nathan Ford for comments on an earlier draft of this manuscript.

***Financial support.*** This work was supported by the Bill and Melinda Gates Foundation via a grant to the HIV Modelling Consortium (OPP1084364) and a grant on misclassification errors in routine HIV rapid testing (OPP1131208). The Brocher Foundation funded the *Meeting on the social, public health, human rights, ethical and legal implications of the misdiagnosis of HIV status*. C. J. received institutional grant support from the National Institutes of Health [5 R01 HD053268]. S. G. received institutional grant support from UNAIDS, BMGF, and Wellcome Trust, and travel support from the WHO. J. E. received institutional grant support from the Bill and Melinda Gates Foundation.

***Potential conflict of interests.*** All authors: No reported conflicts of interest. All authors have submitted the ICMJE Form for Disclosure of Potential Conflicts of Interest. Conflicts that the editors consider relevant to the content of the manuscript have been disclosed.
