# Supplementary material for: The Cost of Not Retesting: Human Immunodeficiency Virus Misdiagnosis in the Antiretroviral Therapy “Test-and-Offer” Era
Source: Clin Infect Dis. 2017 Apr 24;65(3):522–5. doi: 10.1093/cid/cix341 (PMC5850410; doi:10.1093/cid/cix341)
Supplement: cost_of_not_retesting_R1_appendix_SUBMITTED [file cix341_suppl_cost_of_not_retesting_r1_appendix_submitted.docx]

**The cost of not re-testing: HIV misdiagnosis in the ART ‘test-and-offer’ era: Supplementary appendix**

*Eaton JW, Johnson CC, Gregson S*

**S1. Model description**

**Table S1. Default model parameters**

| **Parameter** | **Description** | **Default value** | **Range** |
| --- | --- | --- | --- |
| $T$ | Total number tested | 10,000 |  |
| $p$ | True prevalence among testers | 0.01 (low prev.)  0.1 (high prev.) | [0.001-0.05] (low prev.)  [0.03, 0.25] (high prev.) |
| $q$ | RDT sensitivity | 0.99 |  |
| $r$ | RDT specificity | 0.98 | [0.92, 0.99] |
| $\gamma$ | Probability of correlated error between A1 and A2 result (or A2 and A3) | 0.2 |  |
| $\beta$ | Probability of correlated error between first testing algorithm outcome and re-testing A1 | 0.05 | [0, 0.3] |
| $c_{A1}$ | Cost of first RDT | US $8 |  |
| $c_{A2}$ | Cost of subsequent confirmatory RDT (A2/A3) | US $6 |  |
| $c_{RA1}$ | Cost of first RDT during pre-ART initiation re-testing | $c_{A1}$ | [$2, $c_{A1}$] |
| $c_{RA2}$ | Cost of confirmatory RDT during re-testing | $c_{A2}$ | [$2, $c_{A2}$] |
| $c_{ART}$ | Cost of ART per year (fully loaded) | US$450 | [$250, $650] |
| $\delta$ | Annual discount rate | 0.06 |  |
| $e_{ART}$ | Life-expectancy for HIV-negative person after ART initiation | 30 years |  |

Model of HIV testing algorithms

Let $T=\text{10,000}$ be the total number tested. The number of true positive is $P=T\cdot p$ and the number true negative is $N=T\cdot(1-p)$.

Outcomes of A1 rapid diagnostic test (RDT) are determined by the assumed sensitivity $q$ and specificity $r$. The number of true positive testers screened positive by the A1 RDT is $P^{+}=P\cdot q$, and the number of true positive testers screened as negative is ­­$P^{-}=P\cdot(1-q)$. Analogously for true HIV negative testers, $N^{-}=N\cdot r$ and the number screened as positive on the A1 RDT is $N^{+}=N\cdot(1-r)$.

Testers with negative A1 RDTs ($N^{-}$ and $P^{-}$) are classified as HIV negative, while those with positive A1 RDTs ($P^{+}$ and $N^{+}$) proceed to the confirmatory A2 RDT. To capture the possibility of correlated errors between the A1 and A2 RDT due to potential user error or environmental factors, there is a probability $\gamma$ that the outcome of A2 is the same as A1 irrespective of status, such that:

$$N^{++}={\gamma\cdot N}^{+}+{\left( 1-\gamma\right)\cdot N}^{+}\cdot\left( 1-r \right)$$

$$N^{+-}={\left( 1-\gamma\right)\cdot N}^{+}\cdot r$$

$$P^{++}={\gamma\cdot P}^{+}+{\left( 1-\gamma\right)\cdot P}^{+}\cdot q$$

$$P^{+-}={\left( 1-\gamma\right)\cdot P}^{+}\cdot\left( 1-q \right)$$

Those screened HIV negative on A2 ($N^{+-}$ and $P^{+-}$) are classified as HIV-negative. In the two-test strategy, A2 positive testers ($P^{++}$ and $N^{++}$) are classified as HIV positive, with $P^{++}$ being the number correctly diagnosed positive and $N^{++}$ the expected number of misclassified HIV-negative persons.

In the thee-test strategy, $P^{++}$ and $N^{++}$ are referred for a second confirmatory test A3 following the same assumptions as the A2 test:

$$N^{+++}={\gamma\cdot N}^{++}+{\left( 1-\gamma\right)\cdot N}^{++}\cdot\left( 1-r \right)$$

$$N^{++-}={\left( 1-\gamma\right)\cdot N}^{++}\cdot r$$

$$P^{+++}={\gamma\cdot P^{++}+\left( 1-\gamma\right)\cdot P}^{++}\cdot q$$

$$P^{++-}={\left( 1-\gamma\right)\cdot P}^{++}\cdot(1-q)$$

Those with outcomes $P^{+++}$ and $N^{+++}$ are classified as HIV-positive.

In the scenario without retesting, persons classified as HIV-positive by the testing algorithm ($P^{pos}=P^{++}$and $N^{pos}=N^{++}$ for the two-test strategy or $P^{pos}=P^{+++}$and $N^{pos}=N^{+++}$ for the three-test strategy) are presumed to be immediately initiated on lifelong ART under ‘test-and-start’ guidelines. $N^{pos}$ is the number of misclassified HIV-negative persons unnecessarily initiated on treatment.

The WHO serial testing algorithms recommended that the testing algorithm be repeated for testers with discrepant A1 and A2 or A3 test results and classified as inconclusive and retest in 14 days if remain inconclusive. For simplicity, we do not model the repetition of the testing algorithm and classify these persons as HIV-negative. This simplification has a negligible effect on the estimated number misclassified or the estimated testing cost.

Re-testing algorithm model

In the scenario with re-testing before ART initiation, all persons classified as HIV positive by the first application of the serial testing algorithm ( are re-tested before ART initiation using the same serial testing algorithm. Conservatively, we assume that there is a probability $\beta$ of a correlated error between the outcome of the first testing algorithm and the outcome of the re-testing outcome. Thus the number classified in each group by the A1 RDT after first testing are:

$$N_{RT}^{+}={\beta\cdot N}^{pos}+{\left( 1-\beta\right)\cdot N}^{pos}\cdot\left( 1-r \right)$$

$$N_{RT}^{-}={\left( 1-\beta\right)\cdot N}^{pos}\cdot r$$

$$P_{RT}^{+}={\beta\cdot P^{pos}+\left( 1-\beta\right)\cdot P}^{pos}\cdot q$$

$$P_{RT}^{-}={\left( 1-\beta\right)\cdot P}^{pos}\cdot(1-q)$$

Those determined negative by the A1 RDT ($N_{RT}^{-}$ and $P_{RT}^{-}$) are classified as negative, while those classified as positive proceed to the confirmatory A2 RDT:

$$N_{RT}^{++}={\gamma\cdot N}_{RT}^{+}+{\left( 1-\gamma\right)\cdot N}_{RT}^{+}\cdot\left( 1-r \right)$$

$$N_{RT}^{+-}={\left( 1-\beta\right)\cdot N}_{RT}^{+}\cdot r$$

$$P_{RT}^{++}={\beta\cdot P_{RT}^{+}+\left( 1-\beta\right)\cdot P}_{RT}^{+}\cdot q$$

$$P_{RT}^{+-}={\left( 1-\beta\right)\cdot P}_{RT}^{+}\cdot(1-q)$$

In the two-test strategy those tested positive on the A2 RDT ($P_{RT}^{++}$ and $N_{RT}^{++}$) are classified as HIV positive and initiated on ART, with $N_{RT}^{++}$ being the remaining number of misclassified HIV-negative persons.

In the three-test strategy for low-prevalence settings, those tested positive on the A2 RDT receive a second confirmatory test A3:

$$N_{RT}^{+++}={\gamma\cdot N}_{RT}^{++}+{\left( 1-\gamma\right)\cdot N}_{RT}^{++}\cdot\left( 1-r \right)$$

$$N_{RT}^{++-}={\left( 1-\gamma\right)\cdot N}_{RT}^{+}\cdot r$$

$$P_{RT}^{+++}={\gamma\cdot P_{RT}^{++}+\left( 1-\gamma\right)\cdot P}_{RT}^{++}\cdot q$$

$$P_{RT}^{++-}={\left( 1-\gamma\right)\cdot P}_{RT}^{++}\cdot(1-q)$$

The groups $P_{RT}^{+++}$ and $N_{RT}^{+++}$ are classified as HIV-positive by retesting and initiated on ART.

Cost model

The total testing cost is determined by the total number of first and confirmatory tests performed. For the two-test (high prevalence setting) strategy, the total testing cost is

$$C_{T}=T\cdot c_{A1}+\left( P^{+}+N^{+} \right)\cdot c_{A2}.$$

For the three-test strategy, the total testing cost is

$$C_{T}=T\cdot c_{A1}+\left( P^{+}+N^{+}+P^{++}+N^{++} \right)\cdot c_{A2}.$$

The expected cost of ART for misclassified HIV-negative persons $N^{pos}$ is calculated as annual cost of providing ART discounted over the remaining life expectancy from the time of ART initiation:

$$C_{ART}^{N^{pos}}={N^{pos}\cdot c}_{ART}\cdot(1-e^{-\delta\cdot e_{ART}})/\delta$$

In the re-testing scenario, the cost of re-testing under the two-test strategy is

$$C_{RT}=\left( P^{pos}+N^{pos} \right)\cdot c_{RA1}+\left( P_{RT}^{+}+N_{RT}^{+} \right)\cdot c_{RA2},$$

and under the three-test strategy the cost of re-testing is

$$C_{RT}=\left( P^{pos}+N^{pos} \right)\cdot c_{RA1}+\left( P_{RT}^{+}+N_{RT}^{+}+P_{RT}^{++}+N_{RT}^{++} \right)\cdot c_{RA2}.$$

The expected treatment cost for misclassified HIV-negative persons under the re-testing scenario is

$$C_{ART}^{N_{RT}^{pos}}={N_{RT}^{pos}\cdot c}_{ART}\cdot(1-e^{-\delta\cdot e_{ART}})/\delta$$

The expected savings from re-testing is calculated the difference between the testing costs and discounted treatment costs in the baseline scenario compared to the re-testing scenario:

$$\text{expected savings}=\left( C_{T}+C_{ART}^{N^{pos}} \right)-\left( C_{T}+C_{RT}+C_{ART}^{N_{RT}^{pos}} \right).$$

The expected number of years within which re-testing becomes cost saving is calculated by solving for the number of years $t$ after which the expected treatment costs for misclassified persons on ART becomes greater than the expected cost of re-testing, that is

$$C_{RT}=\left( N^{pos}-N_{RT}^{pos} \right){\cdot c}_{ART}\cdot(1-e^{-\delta\cdot t})/\delta.$$

This is solved by

$$t=-\frac{1}{\delta}\cdot log \left( \begin{aligned} 1-C_{RT}\cdot\frac{\delta}{{(N}^{pos}-N_{RT}^{pos}){\cdot c}_{ART}} \\ \end{aligned} \right).$$

**S2. Sensitivity Analysis**

The following figures illustrate the sensitivity analysis of the number of years to recover re-testing costs in averted ART costs for ranges in a number of key model parameters (Table S1). In each figure, points illustrate default parameter values used in baseline model scenario presented in main results.

**Figure S1. Testing strategy misclassification probability.** Results illustrate the number of years to recover re-testing costs in averted ART costs for a range of values for the overall probability of misclassification of an HIV-negative person being tested. Results are generated by varying the specificity of each test from 92% to 99% (baseline value 98% specificity).

**Figure S2. Annual cost of ART.** Figure illustrates number of years to recover re-testing costs for annual ART costs ranging from US$250 to $450 (fully loaded—incorporating commodities, healthcare personnel, supply chain, and above-facility management).

**Figure S3. Cost per RDT at re-testing**

**Figure S4. HIV prevalence.**

**Figure S5. Probability of correlated error between first diagnosis and re-testing.**
